# An Atypical Case of Shiga Toxin Producing-*Escherichia Coli* Hemolytic and Uremic Syndrome (STEC-HUS) in a Lung Transplant Recipient

**DOI:** 10.1155/2019/9465040

**Published:** 2019-04-11

**Authors:** Louis Manière, Camille Domenger, Boubou Camara, Diane Giovannini, Paolo Malvezzi, Lionel Rostaing

**Affiliations:** ^1^Service de Néphrologie, Hémodialyse, Aphérèses et Transplantation Rénale, CHU Grenoble-Alpes, Grenoble, France; ^2^Université Grenoble Alpes, Grenoble, France; ^3^Service de Pneumologie, CHU Grenoble-Alpes, France; ^4^Laboratoire d'Anatomie Pathologique, CHU Grenoble-Alpes, France

## Abstract

We herein describe the first case of thrombotic microangiopathy (TMA) which was related to Shiga toxin producing-*Escherichia Coli* Hemolytic and Uremic Syndrome (STEC-HUS) after lung transplantation. His maintenance immunosuppression relied on tacrolimus plus mycophenolic acid. TMA was treated with plasma exchanges (PE) (fresh frozen plasma substitution). After five days of PE, platelets count and lactate dehydrogenase level normalized, whereas hemoglobin continued to gradually decrease and no improvement in kidney function was observed. After seven PE sessions, all TMA biological signs resolved. However, kidney function did not improve, and the patient still required chronic dialysis.

## 1. Introduction

Thrombotic microangiopathy (TMA) is a rare but serious disease. Main causes in naive adult patients are represented by secondary TMA (including pregnancy, malignancies, infections, and drugs) far ahead of primary TMA (Thrombotic Thrombopenic Purpura (TTP)), atypical Hemolytic Uremic Syndrome (aHUS) due to acquired or congenital complement pathway dysregulation and Shiga toxin producing-*Escherichia Coli* Hemolytic and Uremic Syndrome (STEC-HUS) [1–3]. However more specific causes have been described in solid organ transplant recipients, especially in lung transplant recipients. A recent review describes a total of 63 cases of TMA after lung transplantation [4]. Most of the cases are reported during the year following transplantation [4]. For half of the cases, TMA is associated with a concurrent disease, mainly infection [4, 5]. The use of antibiotics, especially those who interact with calcineurin inhibitors (CNI), also contributes to TMA pathogenesis [4, 6]. In addition, immunosuppressive therapy plays a key role [5]. The association of mammalian target of rapamycin inhibitors (mTORi) and CNI as immunosuppressive regimen appears to be the strongest risk factor for TMA after lung transplantation [4, 7, 8]. In a few cases, an acquired deficiency in von Willebrand factor-cleaving metalloprotease (ADAMTS13) has been described [9]. We describe here the first case of TMA due to Shiga toxin producing-*Escherichia Coli* Hemolytic and Uremic Syndrome (STEC-HUS) after lung transplantation.

## 2. Patients and Methods

A 43-year-old man underwent double monopulmonary transplantation for respiratory failure secondary to cystic fibrosis 8 years ago. An acute graft rejection occurred 1 month after his transplantation and was successfully treated with steroids. Thereafter his maintenance immunosuppressive regimen included tacrolimus and mycophenolic acid. His last annual check-up found a normal kidney function with a serum creatinine of 82 *μ*mol/L, i.e., an estimated glomerular filtration rate (eGFR) of 87 mL/min/1,73m^2^ (MDRD estimate), proteinuria on urine sample was at 0.24 g/L and the tacrolimus trough level was not overdosed (6 *μ*g/L). Assessment of donor-specific alloantibody (DSA) by Luminex™ was negative and there was no cytopenia. The patient had been vaccinated against influenzae two months before.

Five days after a complete normal check-up in the pulmonology department, the patient presented with diarrhea and abdominal pain which brought him to emergency department. The patient had low blood pressure and tachycardia and received one liter of intravascular saline 0.9%. He also suffered from mental confusion, diplopia, and anuria. He had no fever and his abdomen was flexible. The biology found an acute kidney injury (AKI) (serum creatinine was 560 *μ*mol/L and blood urea nitrogen 28 mmol/L) without acid-base and electrolyte disturbances. There was also an inflammatory syndrome with a C-reactive protein at 138 mg/L and a procalcitonin at 6.24 *μ*g/L. The blood cell count found leukocytosis at 27.2 G/L (with 80% neutrophils), thrombocytopenia at 58 G/L, and a subnormal hemoglobin level at 125 g/L. There was also a mild hepatic cytolysis (AST 122 UI/L, ALT 108 UI/L) and a low prothrombin rate at 29%. Lactate dehydrogenase (LDH) was elevated at 1.555 UI/L, haptoglobin was at its lower limit (0.5 g/L), but assessment of schistocytes was negative. The abdominal CT-scan revealed nonspecific ileitis and colitis.

The first hypothesis was abdominal sepsis, with AKI secondary to acute tubular necrosis. The patient was admitted in the intensive care unit (ICU) and underwent orotracheal intubation for neurologic failure, continuous hemodialysis for AKI, and anti-infectious therapy including metronidazole and acyclovir. The brain CT-scan found multiple white matter lesions. Initially no germ was found in blood, cerebrospinal fluid, and stools. Assessment of antineutrophil cytoplasmic, antinuclear, and antiphospholipid autoantibodies was also negative.

After five days of symptomatic treatment, there was no clinical improvement; biological tests eventually found the presence of schistocytes (3%). Platelets stabilized at around 60 G/L and hemoglobin progressively decreased to 66 g/L. A complete panel of TMA workup was sent out including complement exploration (classic and alternative pathways, regulation proteins), ADAMTS-13 exploration (activity and antibodies) and Shiga toxin research by* Polymerase Chain Reaction* (PCR) on rectal biopsy (see Table 1). Due to the severe neurological dysfunction, daily plasma exchanges (PE) were begun.

After five days of PE, platelets count and LDH level normalized, whereas hemoglobin continued to gradually decrease and no improvement in kidney function was observed. Complement exploration was normal, ADAMST-13 activity was decreased, but not absent and autoantibodies were negative. Shiga toxin PCR came back positive on rectal biopsy, confirming the Shiga toxin producing-*Escherichia Coli *Hemolytic and Uremic Syndrome (STEC-HUS) (all investigational results are represented in Table 1). After seven PE sessions, all TMA biological signs resolved, and the patient could be extubated. However, kidney function did not improve, and the patient still required dialysis. Proteinuria was massive (around 6.32 g/L). A kidney biopsy was performed nine weeks after admission and found severe cortical atrophy with ischemic impairment on all areas and severe fibrosis and arteriolar hyalinosis related to long exposure to CNI therapy for pulmonary transplantation. He never recovered from AKI.

Chronic hemodialysis was continued, and the patient underwent living-related kidney transplantation (KT) eighteen months later. We did not observe any TMA recurrence after KT, whereas immunosuppression was still based on tacrolimus and mycophenolic acid. The patient developed two months after his KT a cytomegalovirus primary infection, but outcomes were good. Eighteen months after kidney transplantation (KT) serum creatinine was 145 *μ*mol/L.

## 3. Discussion

We describe here the first case of STEC-HUS—one of the most unlikely TMA cause in this case—in a lung transplant recipient with severe neurological and renal involvements. Before its initial presentation and notably the seriousness of neurologic involvement but also the context (solid organ transplant recipient), TTP, aHUS or CNI toxicity would have appeared more credible etiologies. Because of its seriousness, the implementation of plasma exchanges was, in our opinion, fully justified and was associated with neurological recovering without renal function improvement. On the therapeutic side, antibiotics use in STEC-HUS has not yet shown its benefit and remains controversial [3]. Had our patient not improved his neurological condition under plasma exchanges, this could have questioned the use of Eculizumab or immunoadsorption, because these therapies had been successfully used in some cases of STEC-HUS with neurological involvement [10–13].

## 4. Conclusion

STEC-HUS might occur in calcineurin inhibitor-treated solid organ transplant recipients.

## Figures and Tables

**Table 1:** TMA workup and results.

Exams	Results
*Immunology*	
Complement	
C3, mg/L (N: 880-1650)	**780**
C4, mg/L (N: 100-380)	107
CH50, % (N: 86-126)	164
Factor H, % (N: 70-130)	**61**
Antifactor H antibodies, UI (N: 0-300)	< 5
Factor I, % (N: 70-130)	97
ADAMTS13	
Activity, % (N: 40-130)	**22**
Anti-ADAMTS-13 antibodies, UI (N < 15)	< 1.0
Antineutrophil Cytoplasmic Antibodies (N < 20)	< 20
Antinuclear Antibodies (N < 160)	< 160
Antiphospholipid Antibody Syndrome	
Anticardiolipin antibodies	negative
Anti-*β*2GP1 antibodies	negative

*Infectiology*	
PCR Shiga toxin (rectum biopsy)	*positive*
PCR Cytomegalovirus (blood and CSF)	negative
PCR Epstein-Barr Virus (blood, in log cop/mL)	2.8
HIV, HBV, HCV, HEV, *Yersinia*, *Leptospira*, Syphilis, *Coxiella*, Lyme serologies	negative

TMA, thrombotic microangiopathy; CSF: cerebrospinal fluid; PCR: polymerase chain reaction.

## Data Availability

Data availability is possible upon request.
